# Older adults’ outdoor walking and the built environment: does income matter?

**DOI:** 10.1186/s12889-015-2224-1

**Published:** 2015-09-10

**Authors:** M. Winters, R. Barnes, Scott Venners, N. Ste-Marie, H. McKay, J. Sims-Gould, MC Ashe

**Affiliations:** Centre for Hip Health and Mobility, The University of British Columbia, Vancouver, BC Canada; Faculty of Health Sciences, Simon Fraser University, Blusson Hall Rm 11522, 8888 University Drive, Burnaby, BC V5A 1S6 Canada; Department of Family Practice, Faculty of Medicine, The University of British Columbia, Vancouver, BC Canada

## Abstract

**Background:**

Our aim was to examine the association between Street Smart Walk Score® and self-reported outdoor walking among older Canadians, and to determine whether socioeconomic status modifies this association.

**Methods:**

We linked objective walkability data with cross-sectional survey data from the Canadian Community Health Survey Healthy-Aging 2008–2009 Cycle for a sample of 1309 British Columbians aged ≥ 65 years. We examined associations between Street Smart Walk Score and meeting physical activity guidelines (≥150 min of moderate to vigorous activity/week) through self-reported outdoor walking using multivariable logistic regression, and tested for significant interactions with household income.

**Results:**

A ten point higher Street Smart Walk Score was associated with a 17 % higher odds of meeting physical activity guidelines through walking outside (95 % CI: 1.07,1.27). In addition, older adults living in neighbourhoods categorised as *Walker’s Paradise* were over three times more likely to meet guidelines than those living in *Car-dependent/Very car dependent* neighbourhoods. We found no evidence that household income moderated the effect of Walk Score on walking outside.

**Conclusions:**

Neighbourhood design may be one avenue whereby physical activity levels of older people can be enhanced through outdoor walking, with benefit across socioeconomic strata.

## Background

Our society is aging. By 2050, 30 % of people living in North America will be over the age of 60 [1]. Notably, chronic diseases and physical inactivity have been declared global health crises. Chronic diseases were responsible for nearly two-thirds of deaths [2] and physical inactivity was the fourth leading cause of death worldwide [3]. Together these trends have created ‘a perfect storm’ that highlights the need to shift from a disease management model to a model that promotes active healthy aging. At present, the older adult population is highly inactive [4, 5]. Canadian guidelines recommend 150 min of moderate to vigorous physical activity per week [6], which may be equivalent to 7000–10,000 steps per day for older adults [7]. Only 44 % of older Canadians self-report meeting guidelines [4], and when physical activity is measured objectively (using accelerometry), just 13 % meet guidelines [5].

Most older adults wish to grow old in the communities they live in [8, 9]. Recognizing this, the World Health Organization’s Age-friendly Cities Guide recommended creating physical and social environments that offer amenities that support healthy aging [10]. Features of the built environment have been linked to physical activity and health in older adults [11–15], with more walking and better health outcomes associated with more walkable environments [16–20]. Thus developing walkable communities is an opportunity to enhance support for community-based physical activity (e.g., outdoor walking) and could support older adults aging in place.

The diverse array of walkability metrics [21–25] that have been used by researchers makes comparisons across studies challenging. There has been a recent shift toward Walk Score® (www.walkscore.com), a publicly available metric with growing coverage globally. Walk Score is based on proximity to nine destination types (including parks), and is correlated with traditional walkability indices [26–29]. Cross-sectional [17, 30, 31] and longitudinal [32] studies have reported significant associations between Walk Score and walking among adults. With wide geographic availability and consistency, Walk Score is a suitable metric for use in neighbourhood health studies.

Mounting evidence shows the influence of the built environment on physical activity varies across different demographic groups, including by age [16, 33], ability to drive [34] and socio-economic status [29, 35], yet few built environment studies have assessed vulnerable older adults, such as those with low incomes [36, 37]. Older adults of lower socioeconomic status are of poorer health generally [38] and fewer meet physical activity guidelines [39]. Socioeconomic status also impacts travel behavior, with older adults with lower incomes more reliant on active forms of transport [9, 39], potentially related to (lack of) vehicle ownership. Given the vulnerabilities of this aging demographic and the implications for mobility and health, there is a clear need to better understand the influence of the built environment across socioeconomic status.

Therefore, in this study we aimed to address gaps in the literature with a view to understanding if walkability, as measured by Street Smart Walk Score, is associated with walking in older adults. We examined (1) the association between walkability and meeting physical activity guidelines through overall outdoor walking, and (2) whether household income was a moderator of this association.

## Methods

### Data

We used the Canadian Community Health Survey (Healthy Aging cycle) (CCHS-HA) (2008/2009). The CCHS-HA is a cross-sectional survey of *N* = 30,865 adults aged 45 years and older residing in private dwellings in the 10 provinces across Canada [40]. The three Canadian territories were excluded. Informed consent was obtained from respondents, and data collected by computer assisted personal interviewing. Interviews took place from December 2008 to November 2009 achieving an overall response rate of 74 % [40]. For this study we included residents aged 65 years and older living in Census Metropolitan Areas (CMAs) in British Columbia (BC) (Vancouver, Abbotsford-Mission, Kelowna, and Victoria). A CMA is a large census-defined geography, formed by one or more adjacent municipalities centred on a population centre (known as the core), and must have a total population of at least 100,000 of which 50,000 or more must live in the core. This geographical restriction was to limit to respondents living in areas near large urban centres (68 % of British Columbian CCHS respondents 65 years and older lived in CMAs), as relationships between walkability and walking may differ in rural settings [41]. This study did not require ethics review as it was deemed exempt under the *Tri-Council Policy Statement: Ethical Conduct for Research Involving Humans (TCPS 2)*, Article 2.4, by Simon Fraser University’s Office of Research Ethics.

### Measures

#### Walking outside

In the CCHS-HA survey, respondents were asked “over the previous 7 days, how many days did you walk outdoors for any purpose?” with pre-coded response categories for frequencies: never, seldom (1 to 2 days), sometimes (3 to 4 days) and often (5 to 7 days). Those who walked at least 1 to 2 days were then asked, on average, how many hours per day they spent walking (response categories: less than 30 min; 30 min - < 1 h; 1 h - <2 h; 2 h - < 4 h; ≥4 h). We calculated the total minutes walking outside by multiplying frequency and duration of walking trips in the past 7 days, using the midpoint of each response category to estimate days walking and minutes walked per day. For example, we used 15 min as the estimate for those who reported less than 30 min, and 3.5 days for those reporting 3 to 4 days. We derived a binary outcome variable to compare those who did and did not meet current guidelines [6] through outdoor walking by dichotomising the total minutes using a cut point of ≥150 min/week.

#### Street Smart Walk Score

We used the Street Smart Walk Score (generated on August 21, 2013) as a measure of the walkability of respondents’ home environments. We purchased data for the 4527 dissemination areas (census areas with populations of 400–700) within CMAs in BC, using the latitude and longitude of the population-weighted centroid point of each dissemination area. No participant information was sent to Walk Score.

We used the Street Smart Walk Score version of the Walk Score, as it incorporates measures of street connectivity that reflect empirical research [23, 41] and uses the street-network distances rather than straight-line distance to a range of types of amenities including schools, shops, restaurants, parks and cinemas. The scores for each amenity type are summed and normalised to yield a score from 0 to 100. The traditional Walk Score (straight-line distance) has been validated in the US [26–28] and Canada [29], and the Street Smart Walk Score is highly correlated with the traditional Walk Score [31].

We recoded the continuous Street Smart Walk Score into the developer’s categories: 0 to 24 as ‘Very car-dependent’, 25 to 49 as ‘Car-dependent’, 50 to 69 as ‘Somewhat walkable’, 70 to 89 as ‘Very walkable’ and 90 to 100 into ‘Walker’s paradise’. Where the Street Smart Walk Score was not available (2.9 % of respondents), we substituted the traditional Walk Score, based on the developer’s advice (personal communication with Aleisha Jacobson, Business Development Manager, Walk Score, Dec 10, 2014).

#### Household income

All CCHS-HA respondents were asked about the total household income (pre-tax, all sources) in the past 12 months. The categories for household income were in $5000 increments under $20,000; $10,000 increments between $20 and 99,999; and then a category from $100,000 to $149,000 and the upper category above $150,000. To test if household income was a significant moderator of the effect of walkability on walking outside, we collapsed annual household income categories into two groups at using a cutpoint of ≥ $30,000 based on the median family income for Canadian older adults (Statistics Canada, CANSIM table 202–0605). We removed respondents with missing income data (22.4 %) from moderation analysis.

#### Demographic variables

Demographic variables included were respondent sex (male or female), age (in years), highest level of education (less than secondary, secondary/some post-secondary or post-secondary), country of birth (Canada or other), marital status (married/common law or single/widowed/divorced/separated), living alone (yes or no), retirement status (retired or not), BMI (underweight, normal, overweight or obese), chronic conditions (none or one or more), Health Utilities Index (HUI) Mobility Scale (from Level 1 ‘able to walk around the neighbourhood without difficulty and without walking equipment’ through to Level 6 ‘unable to walk’) and fear of falling (yes or no).

### Statistical analysis

Data matching and analyses took place within the Statistics Canada Research Data Centre at Simon Fraser University, Burnaby, British Columbia. We joined the Street Smart Walk Score data to the CCHS-HA by identifying the dissemination areas for all postal codes, and matching the Walk Score data to survey responses by postal code. Of the 6504 BC respondents to the CCHS-HA, 1384 were aged 65 years or more and resided in the CMAs in BC. We removed 16 respondents who had a Health Utilities Index mobility score which indicated that they could not walk at all (Level 6) and a further 59 respondents with missing demographic or outcome data, resulting in a final sample size of *n* = 1309, representing 362,365 British Columbian residents. All statistical analyses were conducted in SAS, version 9.4 (Cary, NC). We calculated frequencies using PROC SURVEYFREQ, and means and standard deviations using PROC SURVEYMEANS. We weighted demographic frequencies using the Statistics Canada proportional sampling scheme and applied Balanced Repeated Replication (BRR) with 500 bootstrap weight variables. We used logistic regression (PROC SURVEYLOGISTIC) to identify the odds of meeting physical activity guidelines through walking outside for each additional point in Street Smart Walk Score; and in separate models looking at walkability category using the ‘Very car-dependent/car dependent’ as reference category (Objective 1), applying the BRR weighting method. We included potential confounders of sex, age, education, country of birth, HUI mobility scale and fear of falls in the final models. We offered potential confounders including sex, age, education, country of birth, HUI mobility scale, and fear of falls, to the final multivariable models. We retained those variables that remained significant (*p* < 0.05), as well as age and sex. To address the second objective to examine whether income was a moderator of the effect of the walkability on meeting physical activity guidelines, we added an interaction term between household income and the Street Smart Walk Score.

## Results

### Sample characteristics

More than half of our sample were women and the mean (SD) age was 75 (8.3) years (Table 1). Close to 50 % of our sample had a post-secondary degree or diploma. While 22 % did not select an income category, of those who did, 0.8 % were in the lowest two categories (<$10,000) and 3.2 % were in the highest category ($150,000 and over). Overall 55 % had an annual household income of ≥ CAN $30,000. The vast majority of respondents had at least one chronic condition (90 %), although 87 % had a mobility score that indicated they could walk around their neighbourhood unaided. Most respondents (93 %) were retired and about a third (35 %) reported a fear of falls. The proportions of weighted populations living in each Walk Score category were 31.0 % in ‘Walker’s paradise’, 35.0 % in ‘Very walkable’, 27.5 % in ‘Somewhat walkable’ and 6.5 % for ‘Very car-dependent/car-dependent’.

**Table 1:** Demographic characteristics of CCHS-HA respondents (≥65 years) (*n* = 1309) living in BC Census Metropolitan areas

Characteristic	Weighted^a^ N	Weighted %/Mean (SD)
Sex		
Male	163,186	45.0
Female	199,178	55.0
Age	362,365	75 (8.3)
Education		
Less than secondary	96,903	26.7
Secondary/Some post-secondary	95,073	26.2
Post-secondary degree/diploma	170,389	47.0
Household income		
< $30,000	84,769	23.4
$30,000 or more	196,595	54.3
Don’t know/Not stated/Refused	81,001	22.4
Country of birth		
Canada	183,431	50.6
Other	178,933	49.3
Marital status		
Married/Common law	237,649	65.6
Single/Widowed/Divorced/Separated	124,715	34.4
Lives alone		
Yes	106,862	70.5
No	255,503	29.5
Retirement status		
Retired	336,271	92.8
Not retired	26,094	7.2
BMI		
Underweight	13,742	3.8
Normal	163,532	45.1
Overweight	112,666	31.1
Obese	41,227	11.4
At least one question not answered	31,197	8.6
Chronic conditions		
None	35,261	9.7
One or more	327,104	90.3
Mobility		
Level 1^b^	315,259	87.0
Level 2/3^c^	35,486	9.8
Level 4/5^d^	11,619	3.2
Fear of falls		
No	236,028	65.1
Yes	126,336	34.9
Walk Score category		
Very Car-dependent/car-dependent	23,592	6.5
Somewhat walkable	99,616	27.5
Very walkable	126,748	35.0
Walker’s paradise	112,408	31.0

SD = Standard deviation

^a^Weighted demographic frequencies using the Statistics Canada proportional sampling scheme and Balanced Repeated Replication (BRR) applied with 500 bootstrap weight variables

^b^Level 1 – able to walk around the neighbourhood without difficulty and without walking equipment

^c^Level 2 – able to walk around the neighbourhood with difficulty but does not require walking equipment or the help of another person; Level 3 – able to walk around the neighbourhood with walking equipment but without the help of another person

^d^Level 4 – able to walk only short distances with walking equipment, and requires a wheelchair to get around the neighbourhood; Level 5 – Unable to walk alone, even with walking equipment. Able to walk short distances with the help of another person and requires wheelchair to get around the neighbourhood

**Table 2:** Odds Ratios (OR) for meeting physical activity guidelines through walking outside

	Model A: Continuous Walk Score^a^		Model B: Categorical Walk Score^a^	
	Unadjusted	Adjusted^b^	Unadjusted	Adjusted^b^
	OR (95 % CI)	OR (95 % CI)	OR (95 % CI)	OR (95 % CI)
Street Smart Walk Score (OR for 10-point change)	1.12 (1.04,1.20)	1.17 (1.07,1.27)	-	-
Street Smart Walk Score Categories				
Very Car-dependent/car-dependent	-	-	1.00	1.00
Somewhat walkable	-	-	1.64 (1.08,2.50)	1.83 (1.16,2.88)
Very walkable	-	-	1.59 (1.05,2.41)	1.95 (1.25,3.07)
Walker’s paradise	-	-	2.98 (1.55,5.72)	3.57 (1.62,7.87)
Sex				
Male	-	1.00	-	1.00
Female	-	0.87 (0.64,1.17)	-	0.87 (0.64,1.17)
Age	-	0.99 (0.97,1.02)	-	0.99 (0.97,1.02)
Education				
Less than secondary	-	1.00	-	1.00
Secondary/some post-secondary	-	1.65 (1.10,2.48)	-	1.63 (1.08,2.45)
Post-secondary graduation	-	1.75 (1.20,2.56)	-	1.76 (1.21,2.58)
Country of birth				
Canada	-	1.00	-	1.00
Other	-	1.15 (0.85,1.55)	-	1.17 (0.86,1.57)
Mobility				
Level 1^c^	-	1.00	-	1.00
Level 2/3^d^	-	0.30 (0.19,0.47)	-	0.30 (0.19,0.48)
Level 4/5^e^	-	0.16 (0.03,0.83)	-	0.16 (0.03,0.86)
Fear of falls				
No	-	1.00	-	1.00
Yes	-	0.81 (0.57,1.14)	-	0.81 (0.57,1.15)

^a^Models weighted using the Statistics Canada proportional sampling scheme and Balanced Repeated Replication (BRR) applied with 500 bootstrap weight variables

^b^Adjusted for sex, age, education, country of birth, mobility and fear of falls

^c^Level 1 – able to walk around the neighbourhood without difficulty and without walking equipment

^d^Level 2 – able to walk around the neighbourhood with difficulty but does not require walking equipment or the help of another person; Level 3 – able to walk around the neighbourhood with walking equipment but without the help of another person

^e^Level 4 – able to walk only short distances with walking equipment, and requires a wheelchair to get around the neighbourhood; Level 5 – Unable to walk alone, even with walking equipment. Able to walk short distances with the help of another person and requires wheelchair to get around the neighbourhood

### Meeting physical activity guidelines through walking outside

**Fig. 1:**
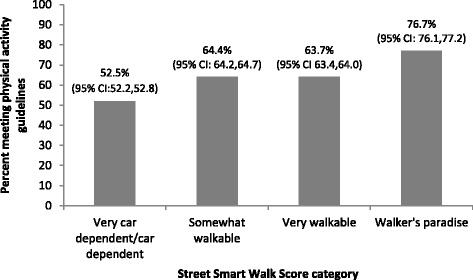
Proportion of Canadian Community Health Survey – Health Aging Cycle respondents (≥65 years) in BC Census Metropolitan Areas (*n* = 1309) meeting physical activity guidelines through walking outdoors, by neighbourhood walkability

Overall, 61.3 % (95 % CI: 61.2,61.5) of older adults met physical activity guidelines through outdoor walking. This outcome varied by setting, with 76.7 % (95 % CI: 76.1,77.2) of respondents who lived in a ‘Walker’s paradise’ acquiring adequate physical activity through their outdoor walking. The proportion meeting guidelines in ‘Very walkable’ neighbourhoods (63.7 %; 95 % CI: 63.4,64.0) was similar to those in ‘Somewhat walkable’ neighbourhoods (64.4 %; 95 % CI: 64.2,64.7), and much higher than for respondents from who lived in ‘Very car-dependent/car-dependent’ neighbourhoods (52.5 %; 95 % CI: 52.2,52.8) (Fig. 1).

In adjusted models (Table 2), a ten-point increase in Street Smart Walk Score was associated with a 17 % increase in the odds of meeting physical activity guidelines through walking outdoors (OR = 1.17; 95 %CI: 1.07,1.27). In the models using the Walk Score categories, respondents living in a ‘Walker’s paradise’ had three and half times the odds of meeting guidelines through walking outside, compared with those living car dependent neighbourhoods (OR = 3.57; 95 % CI: 1.62,7.87). In both models, higher education and greater mobility (by HUI score) were significantly associated with the outcome.

Of those who provided income information, 30 % had household incomes lower than CAN$30,000. Compared to individuals with higher incomes, those with lower incomes were more likely to be male, older in age, retired, living alone, have at least one chronic disease, have a mobility level of 2 or 3, have a less than secondary school education, and not be married or in a common law relationship. The prevalence of lower income varied by CMA but was not significantly different (30.6 % of participants the Vancouver CMA reporting incomes below, 52.1 % in the adjacent Abbotsford CMA, 24.5 % in the Victoria CMA and 34.8 % in the Kelowna CMA; weighted population rounded to the nearest *n* = 50, Pearson chi-square *p* = 0.20). We included interaction terms in the multivariable models to assess any effect modification by income on the association between walkability and meeting physical activity guidelines by walking outdoors. Interaction terms were not significant [Model A (continuous Walk Score), *p*-value = 0.24; Model B (categorical Walk Score), p-values ranged from 0.35 to 0.43].

## Discussion

We extend the current literature by evaluating the association between neighbourhood walkability and physical activity in a population-based sample of older British Columbians. Specifically, our findings build on previous research linking walkability or access to destinations to outdoor walking, specifically in older adults [13–15]. To our knowledge, ours is the first large study to use the Walk Score metric with a focus on the older adult populations. Given the devastating consequences of physical inactivity for older adults (loss of muscle and bone mass, increased obesity, increased prevalence of cancers, diabetes and heart disease [14] and a shortened lifespan [42]), it seems imperative to ensure that neighbourhoods are designed in ways that promote physical activity in this population.

Street Smart Walk Score was positively associated with the odds of achieving sufficient levels of physical activity specifically through outdoor walking. Three in five older adults in our sample reported walking outside for at least 150 min in the previous week, but respondents had over three times higher odds of achieving this if they lived in a ‘Walker’s paradise’ compared with a ‘Car dependent/very car dependent’ neighbourhood. Results did not vary significantly across levels of household income. One previous study of older adults (≥70 years) in Minnesota found no association between Walk Score and physical activity, potentially due to the small sample (*n* = 53) or limited environmental variability [43]. Four studies in adult populations (all ages) found positive associations between Walk Score and walking of similar magnitudes [29–32]. Importantly, the Street Smart Walk Score is publicly available metric with consistent methodology across settings. As a result, our findings can be readily compared with those in other locations, a challenge when walkabilty metrics are study-specific.

Many studies have considered the purpose of walking – whether it is for transportation or recreation Typically, land use mix (destination diversity) and street pattern are more strongly associated with transportation-related walking [25], whereas proximity to parks has been independently associated with recreational walking [44, 45]. We were unable to assess this, as the CCHS-HA questions do not capture purpose. In older populations the distinction between transportation and recreation walking may be blurred, if daily walks are also errand trips. Local evidence shows older adults made fewer than 10 % of trips purely for exercise (travel diary data) [17], suggesting the majority of walking trips do have a utlitarian component. Street Smart Walk Score captures proximity to 13 types of destinations, one of which is proximity to parks. As such, the metric may have more relevance to recreational-related walking than have traditional land use mix and walkability indices. Irrespective of whether outdoor walking by CCHS-HA respondents in our study was for transportation or recreation, those living in more walkable neighbourhoods had far higher odds of partaking in sufficient outdoor walking to achieve health benefit.

Our results illustrate the potential for the built environment to support older adults to walk outside. This study provides further evidence that macroscale design features - connected neighbourhoods with commercial and recreational facilities – may better enable older adults to achieve health-enhancing physical activity levels through neighbourhood walking. Street Smart Walk Score does not explicitly capture detailed design level features, for example presence and condition of sidewalks and benches are not measured. We acknowledge the relevance of microscale built environment [13] and the concept of person-environment fit [11, 46] especially for older adults. Complementary studies have used street audits [16, 47, 48] and photovoice [49] to identify specific design features that help or hinder older adults in traversing their neighbourhoods. Taken together, studies of macroscale and microscale built environment features provide guidance on community design that supports active healthy aging at varying scales.

Our interest was in whether income levels moderated the effect of the neighbourhood environment on walking. Previous work suggests that the built environment has more influence on those with higher incomes, for example, Manaugh and colleagues [29] reported that Walk Score was more strongly associated with walking in wealthy households than lower income households. This may be due to restricted travel options faced by those with lower incomes, or less flexible time for recreational activities. In our study, household income was a not a significant moderator of the association between Walk Score and outdoor walking. This may be an important finding as the built environment has the potential to reduce health inequalities [50], if it serves to promote positive health behaviours, in not only high income by also low income populations. We used household rather than individual income, on the premise that household income is likely a better reflection of the resources available. Since 70 % of CCHS-HA respondents live alone, household income equates to individual income in most cases. A more pressing question may be whether income reflects the overall wealth. Statistics Canada typically asks about income “from all sources” [51], which may not capture wealth related to home ownership or retirement savings. Both of these are of particular relevance for older adults. Neighbourhood income levels may also play a role. For example, King and colleagues [19] recruited older adults who lived in four neighbourhoods types in Seattle and Baltimore: high and low walkable, and high and low income. Participants in high walkability, high income neighbourhoods were most likely to meet physical activity guidelines. We were unable to evaluate such interactions using the CCHS-HA.

The use of administrative data introduces some limitations. First, the CCHS-HA survey uses self-reported measures, subject to social desirability and recall error that may result in overestimates of physical activity. This would not likely vary across built environment settings, directing any bias toward the null. Recently objective measures of physical activity have been incorporated into a Canadian national survey [52] with a smaller sample size. As with any survey question related to income, we had substantial missing data for income (22.4 % of respondents, more likely to be female). We excluded these respondents from the effect modification analysis, which may have introduced bias if those who do not answer the income question are not evenly distributed across income levels. Studies have found that those who refuse are likely to be from higher occupational positions, whereas those who respond ‘don’t know’ are likely to be from lower occupational status [53, 54]. In our data, we found no significant differences for education and retirement status (other indicators of socio-economic status) between those with and without income data, and likewise no differences in Walk Score. Second, perceptions of the built environment were not captured in the CCHS-HA. The survey did not include questions that allow adjustment for self-selection (that people who like to walk may choose to live in more walkable neighbourhoods), although emerging evidence supports a role for the built environment independent of people’s preferences for walking [55–57]. Third, the CCHS-HA had a question on pet ownership, but not specifically dog ownership; the literature suggests that it is dog ownership that is associated with walking [58, 59]. Fourth, survey data were collected in 2008–2009 and the Walk Score data were from 2013. This is of little concern given the slow nature of changes to the built environment. Finally, we conducted analysis of cross-sectional data, thus results should not be taken as causal, but rather they provide the backdrop to design and implement longitudinal studies [20].

Our study also had a number of strengths. We used a large, national survey of older adults (CCHS-HA) that provided a representative sample across a broad geographical area. Further, we used Street Smart Walk Score, a comparable, objective measure of walkability to allow our results to be placed into context. We were able to explore potentially moderating effects of income given the large sample size.

## Conclusion

Older adults are at greater risk of deleterious health effects associated with a physically inactive lifestyle than younger adults. Neighbourhood design may be one avenue whereby physical activity levels of older people can be enhanced through outdoor walking, and this may provide population-level impacts. We found neighbourhood walkability was strongly associated with older adults’ outdoor walking, with benefits across socioeconomic strata. Be it through utilitarian walking to complete daily errands, recreational walking to destinations such as parks, or a combination of both, our findings highlight the opportunity to promote outdoor neighbourhood walking and through this, to improve the health of older adults.

## Abbreviations

BC: British Columbia
CCHS: Canadian Community Health Survey
CMA: Census Metropolitan Areas
CCHS-HA: Canadian Community Health Survey- Healthy Aging
CI: Confidence interval
HUI: Health Utilities Index
